# Sex‐ And tissue‐specific differences in telomere length in a reptile

**DOI:** 10.1002/ece3.5164

**Published:** 2019-05-22

**Authors:** Nicky Rollings, Christopher R. Friesen, Camilla M. Whittington, Rasmus Johansson, Richard Shine, Mats Olsson

**Affiliations:** ^1^ School of Life and Environmental Sciences University of Sydney Sydney New South Wales Australia; ^2^ School of Biological Sciences University of Wollongong Wollongong New South Wales Australia; ^3^ Sydney School of Veterinary Science University of Sydney Sydney New South Wales Australia; ^4^ Department of Biological & Environmental Sciences University of Gothenburg Gothenburg Sweden; ^5^ Department of Biological Sciences Macquarie University Sydney New South Wales Australia

**Keywords:** *Ctenophorus pictus*, life history, painted dragon lizard, reptiles, sex differences, telomeres

## Abstract

The usage of telomere length (TL) in blood as a proxy for the TL of other tissues relies on the assumption that telomere dynamics across all tissues are similar. However, telomere attrition can be caused by reactive oxygen species (ROS) which may vary with metabolic rate, which itself varies across organs depending upon the life history strategy of an organism. Thus, we chose to measure the telomeres of various cell types in juvenile painted dragon lizards, *Ctenophorus pictus*, given their unusual life history strategy. Individuals typically only experience a single mating season. We measured the TL of male and female dragons using qPCR and observed that TL varied with tissue type and sex. Telomeres of blood cells were longer than those of liver, heart, brain, and spleen, and females had longer telomeres than males. Brain telomeres in males were approximately half the length of those in females. Telomeric attrition in the male brain may be due to the need for rapid learning of reproductive tactics (territory patrol and defense, mate‐finding). Significant correlations between the TL of tissue types suggest that blood TL may be a useful proxy for the TL of other tissues. Our comparison of organ‐specific telomere dynamics, the first in a reptile, suggests that the usage of blood TL as a proxy requires careful consideration of the life history strategy of the organism.

## 1 INTRODUCTION

Telomeres, short tandem repeats of TTAGGG located at the ends of chromosomes (Blackburn, 2000; Blackburn & Gall, 1978), are highly dynamic structures that are tightly linked to the life history strategies of species (Gomes, Shay, & Wright, 2010; Haussmann & Marchetto, 2010; Rollings, Uhrig, et al., 2017), and even morphs within species (Rollings, Friesen, et al., 2017). Telomeres are highly dynamic because they are affected by several factors, to a degree which may be determined by the life history strategy of the organism through the allocation of resources (Haussmann & Marchetto, 2010). Every round of cellular division typically shortens telomeres, due to incomplete chromosomal replication (Olovnikov, 1973), but telomeres may be extended again through activity of the enzyme telomerase (Giardini, Segatto, Silva, Nunes, & Cano, 2014; Greider, 1990).

Telomeres may also become shorter when damaged by reactive oxygen species (ROS, Houben, Moonen, Schooten, & Hageman, 2008, Olsson, Friesen, et al., 2018, Selman, Blount, Nussey, & Speakman, 2012, von Zglinicki, 2002). Reactive oxygen species are produced in mitochondria when electrons leak from the electron transport chain during oxidative phosphorylation and interact with molecular oxygen (Beckman & Ames, 1998; Turrens, 2003). As oxidative phosphorylation produces most of an organism's ATP (Bertram, GRAM PEDERSEN, M., LUCIANI, D. S., & SHERMAN, A., 2006), an increase in metabolism may increase ROS production and cause oxidative stress. However, ROS may be countered by antioxidants (Magwere et al., 2006; Monaghan, Metcalfe, & Torres, 2009), reducing telomeric attrition. Organisms that prioritise reproduction or growth over cellular maintenance (such as antioxidant production) often age more quickly, have shorter life spans, and experience higher rates of telomere loss (Promislow & Harvey, 1990; Ricklefs & Wikelski, 2002). Metabolic rates may vary between organs and tissues (and are frequently correlated with organ size, Piersma, Gudmundsson, & Lilliendahl, 1999, Wang et al., 2010), often influenced by life history strategy (Ricklefs & Wikelski, 2002). Thus, ROS concentrations may also vary among tissues, and tissues or organs may experience unique telomere dynamics, depending upon the life history strategy of the organism. We would predict that organs with higher oxidative stress would experience higher rates of telomeric attrition.

Many studies of telomeres, particularly those in evolutionary biology, focus on telomere lengths (TL) in whole blood or white blood cells (Badas et al., 2015; Barrett & Richardson, 2011; Bize, Criscuolo, Metcalfe, Nasir, & Monaghan, 2009; Froy et al., 2017; Lopez‐Arrabe et al., 2018; Olsson, Wapstra, & Friesen, 2018; Rollings, Uhrig, et al., 2017). This focus on blood is largely due to ease of collection, relatively low invasiveness, and because it allows repeated collection of samples. However, without knowing whether blood TL are representative of the various organs and tissues within an organism, it is difficult to determine whether blood TL provides meaningful inferences about the organism as a whole.

Research on zebra finches has shown robust correlations between telomere dynamics in red blood cells and the brain, liver, and spleen (Reichert, Criscuolo, Verinaud, Zahn, & Massemin, 2013). Telomere lengths correlate between tissues in humans, dogs, and pigs (Benetos et al., 2011; Daniali et al., 2013; Fradiani, Ascenzioni, Lavitrano, & Donini, 2004; Friedrich et al., 2000), although a study of human cadavers testing a wide range of tissues found few such correlations (Dlouha, Maluskova, Lesna, Lanska, & Hubacek, 2014). Telomere lengths are also consistent between tissues in zebrafish (Lund, Glass, Tolar, & Blazar, 2009), whereas a study on Wistar rats reported shorter telomeres in males than females in kidneys, liver, pancreas, and lungs, but not brain (Cherif, Tarry, Ozanne, & Hales, 2003). This study also reported telomeric attrition with age in all tissues for both sexes except for the brain. Thus, while correlations between cell types have been observed, research has focussed on a limited range of taxa. In order to determine whether trends of telomere correlation are consistent across a broader range of taxa, in particular those with unusual life history strategies, we designed this study on a short‐lived Australian lizard.

**Figure 1:**
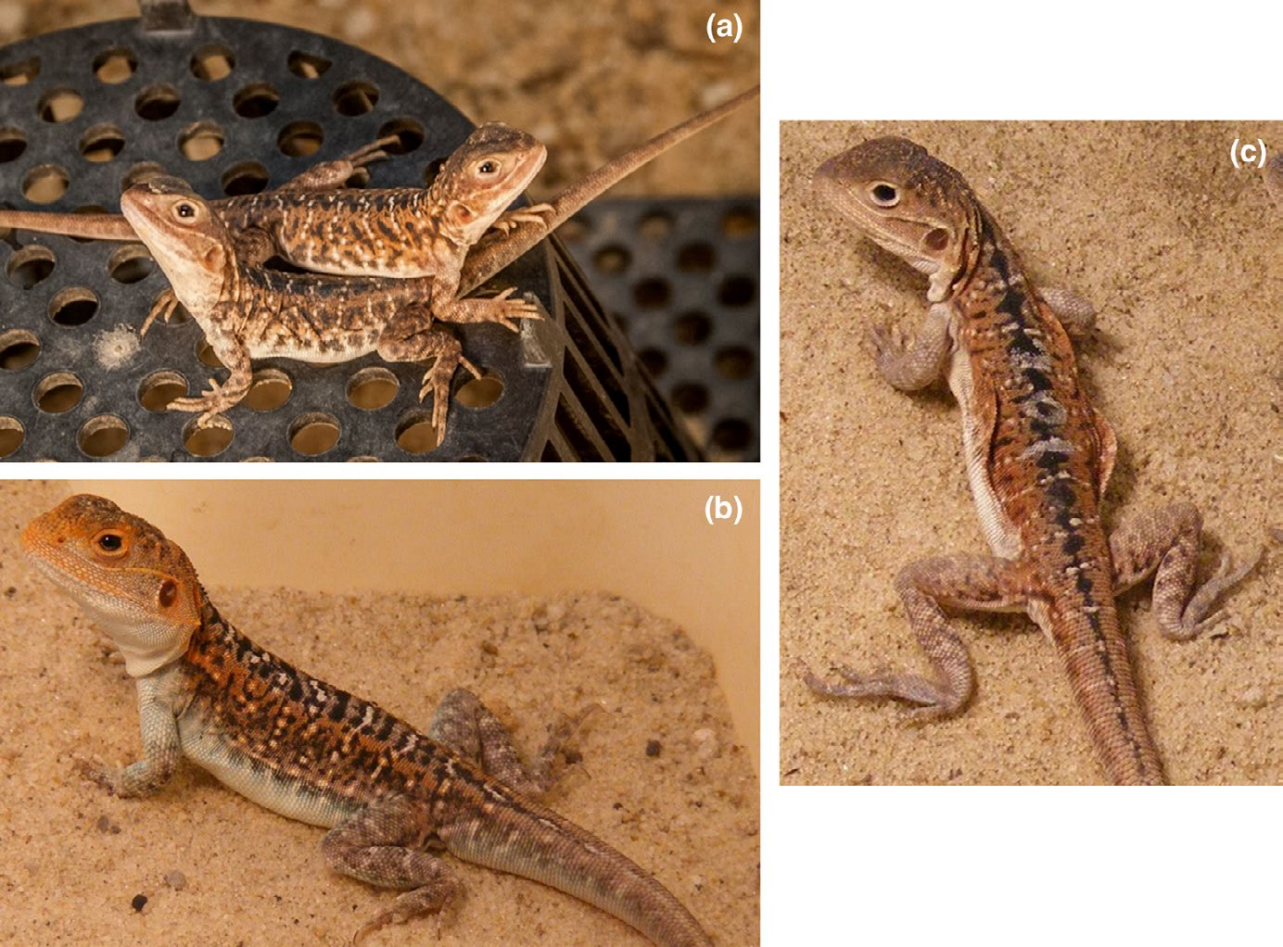
Plate of examples of *Ctenophorus pictus*. a) Juvenile dragons. b) Adult male. c) Adult female (the abdominal skin flaps on the female indicate recent oviposition)

We assessed TL across a range of tissues in juvenile painted dragon lizards, *Ctenophorus pictus*, a reptile species with a notable life history strategy (Figure 1). These dragons are short‐lived (~1 year in the wild, Olsson, Healey, Wapstra, et al., 2007) agamid lizards found in semiarid regions in the southern half of Australia. Males are polymorphic in color (red, orange, yellow, and a lack of head color referred to as “blue,” Healey, Uller, & Olsson, 2007, Olsson, Healey, & Astheimer, 2007, Olsson, Schwartz, Uller, & Healey, 2009, Rollings, Friesen, et al., 2017) and exhibit morph‐specific adult behaviors as they patrol territories and court females. By contrast, females are monomorphic, sedentary, and cryptic (Olsson, Healey, Wapstra, et al., 2007). Territories of individual males may be up to 50 m in length and are patrolled to prevent ingress by neighboring or invading males (Healey et al., 2007). Most dragons only participate in a single mating season, thus males must rapidly learn the behaviors of neighboring males and understand the structures of their territories to increase their chances of successful reproduction. Therefore, their ability to process such information is likely under strong selection. The importance of effective patrolling may be reflected in brain structure, with a comparatively larger optic tectum in the painted dragon than in other closely related species (Hoops, Vidal‐García, et al., 2017). The tectum, the primary processing center for visual information (Bischof & Watanabe, 1997), is involved in processing agonistic visual displays perceived in signaling (McDonald, Paul, & Hawryshyn, 2004), and thus may be important for observation and patrol of a territory. Male painted dragons also have a larger medial preoptic nucleus (MPON) than do conspecific females (Hoops, Ullmann, et al., 2017). The MPON facilitates male reproductive behavior, such as mate‐seeking and aggression (Balthazart & Ball, 2007; O'Connell & Hofmann, 2011). The sex difference in MPON size suggests that brain function is important in male dragons for successful reproduction. Territorial behaviors among males begin before reaching maturity (Healey et al., 2007), with morph type influencing the probability of winning contests, suggesting that the brain develops before other reproductive structures. As females do not patrol or maintain territories, it is likely that they do not need to devote resources to rapid brain development. This species is less sexually dimorphic in body size than are most other *Ctenophorus* species (Hoops, Ullmann, et al., 2017), and the sex of juvenile dragons is indistinguishable (without inspection for hemipenes) until males develop colors as they approach maturity. The lack of size dimorphism suggests that males and females invest similarly in growth rates, and thus the cost of rapid brain development in the males is likely paid elsewhere. Males may prioritise growth and brain development over cellular maintenance, possibly downregulating antioxidant production and causing higher oxidative stress, which may increase telomere attrition. We, therefore, predict that juvenile male dragons will have shorter telomeres than females, especially in the brain (as it is a site of rapid development). By extension we predict there will be correlations in TL between the tissue types, with potentially the weakest correlation between brain and the other tissues due to the life history strategy of the males described above. Our comparative study of tissue‐ and sex‐specific telomere dynamics, the first in a reptile, investigates telomeres while also considering the internal and external environment of the species.

## 2 MATERIALS AND METHODS

### 2.1 Study organism

Mature (~9 months old) female dragons (*Ctenophorus pictus*, W. Peters, 1866) were caught by noose or hand at Yathong Nature Reserve, NSW, Australia (145°35′E; 32°35′S) and taken to holding facilities at the University of Sydney in October 2015 where they were housed for the duration of the experiments. Animals were collected under a permit issued by NSW National Parks and Wildlife Service (SL100352), and experiments were conducted in accordance with University of Sydney ethics approval (AEC‐2013/6050). Females were housed in pairs in opaque plastic tubs (330 × 520×360 mm) with sandy substrate and exposed to a 12 hr light: 12 hr dark cycle. The lizards were fed mealworms and crickets, dusted with calcium and multivitamins, to satiation every day, and the cages were misted with water once a day. Heat lamps and ceramic hides were provided to allow the lizards to thermoregulate to their preferred body temperature (36°C; M.O., unpublished data obtained from cloacal temperature readings in the wild). A mound of moist sand was available in each tub to allow females to burrow and lay eggs. Females were checked for oviposition each day, as apparent from skin flaps around the abdomen. Overall, 18 females produced 22 clutches and 89 eggs in total between 4 November 2015 and 6 January 2016. The eggs were removed and weighed to the nearest 0.1 g. As part of an investigation on the effects of temperature on TL, the individual eggs from each clutch were then placed sequentially in one of four incubators (27, 30, 32, 36°C, ±0.5°C) to separate clutch and temperature effects. Eggs were half‐buried in a 1:7 mix of water to vermiculite in sealed, transparent, containers in each incubator. Containers were sealed to reduce evaporation of water but aerated weekly. Eggs were checked daily for nonviable eggs, which were removed. Seventy‐three dragons hatched and 16 nonviable eggs were removed (most from the 36°C incubator, which is warmer than estimated incubation temperature in the wild: approximately 30°C, M.O., unpublished data). Hatchlings were sexed (determined by the presence or absence of hemipenes), weighed to the nearest 0.1 g, and snout–vent length (SVL) and total length were measured to the nearest mm. Each individual was marked with a unique toe‐clip pattern for identification. Individuals were then placed in groups in tubs with the same conditions as the adult females, with the exception of the sand mound. Hatchling dragons were fed pinhead crickets, dusted in calcium and multivitamins, to satiation every day. Mortality is high in juvenile painted dragons but tubs were checked multiple times a day and any dead individuals were promptly removed.

### 2.2 Sample collection

In March 2016, the 24 remaining juveniles (14 female, 10 male, ranging in age from 97 to 140 days, 1 female and 3 males incubated at 27°C, 6 females and 5 males incubated at 30°C, 5 females and 2 males incubated at 32°C, 2 females incubated at 36°C) were euthanized by decapitation. Prior to decapitation individuals were first sedated by cooling in a refrigerator for 10 min and in a freezer for 2 min. They were sexed again to confirm prior assignations, weighed to the nearest 0.1 g, and SVL and total length measured to the nearest mm. Whole blood, whole brain, heart, liver, and spleen were then collected. Each tissue type was placed in 300 µl of RNAlater (Sigma‐Aldrich, Castle Hill, NSW, Australia) and stored at −80°C until DNA extraction.

### 2.3 Quantifying relative telomere length

To analyse relative telomere length (RTL; relative to the 18S gene), we first purified DNA from the collected tissues. The brain, heart, and liver were sliced into small pieces, and 50 µl of the diluted blood was used in the extraction. A DNeasy Blood and Tissue Kit (Qiagen, Chadstone, VIC, Australia) was used for extractions, according to the manufacturer's instructions. The protein kinase digestion step was run for 10 min for the blood, but the brain, heart, liver, and spleen required overnight incubation for complete digestion. RNase A (Qiagen, Chadstone, VIC, Australia) was added at the recommended concentration. The DNA concentration (ng/µl) and A260:A280 ratio of each sample were measured in duplicate using a Nanodrop (Thermo Fisher Scientific) and aliquots diluted to 10 ng/µl using the AE buffer provided in the DNA extraction kit. Only samples with a A260:A280 ratio between 1.7 and 1.9 and a concentration above 10 ng/µl were considered high enough quality to be used in analyses. Ultimately, 22 blood (12 female, 10 male), 18 liver (9 female, 9 male), 17 heart (8 female, 9 male), 16 brain (11 female, 5 male), and 10 spleen (6 female, 4 male) samples were of sufficient quality for use in the study. DNA was stored at −30°C.

Telomere length was measured using real‐time quantitative PCR (qPCR) using SensiMix SYBR No‐ROX Kit (Bioline, Sydney, NSW, Australia) and a Rotor‐gene 6000 thermocycler (Qiagen, Chadstone, VIC, Australia) according to published protocols (Rollings, Friesen, et al., 2017; Rollings, Uhrig, et al., 2017) using techniques developed by Criscuolo et al. (2009) and Plot, Criscuolo, Zahn, and Georges (2012) with the 18S ribosomal RNA (18S) gene as the nonvariable copy number reference gene. The telomere primers used were Telb1 (5′‐CGGTTTGTTTGGGTTTGGGTTTGGGTTTGGGTTTGGGTT‐3′) and Telb2 (5′‐GGCTTGCCTTACCCTTACCCTTACCCTTACCCTTACCCT‐3′, Cawthon, 2002).

The 18S gene (92 bp amplicon in *Anolis*) was selected as the reference gene as it had previously been validated in reptiles (Plot et al., 2012; Rollings, Uhrig, et al., 2017). The primer sequences used were 18S‐*F* (5′‐GAGGTGAAATTCTTGGACCGG‐3′) and 18S‐R (5′‐CGAACCTCCGACTTTCGTTCT‐3′). Reactions were run in triplicate for each sample, with each run containing either Telb or 18S primers. Amplifications were carried out in a Rotor‐Gene 6000 thermocycler (Qiagen, Australia) using an initial Taq activation step at 95°C for 10 min and a total of 40 cycles of 95°C for 15 s, 60°C for 15 s, and 72°C for 15 s. Each reaction had a final volume of 20 µl with 10 ng of DNA, forward and reverse primers used at a concentration of 250 nM, and MgCl_2_ added for a concentration of 1.7 mM. 11.25 µl of the SensiMix SYBR No‐ROX Master Mix was added per reaction. A melt curve was generated after each run over the temperature range of 60 to 95°C to ensure that there was no nonspecific product amplification (see appendix Figure S1 and Figure S2 for examples). All of the DNA samples for a given individual were included in the same run. No‐template control reactions were run in triplicate for each primer set during every qPCR run to ensure that there was no contamination. Standard curves were produced, using the pooled DNA from three randomly selected lizards, for both telomeres and 18S using fourfold serial dilutions to ensure consistent rates of amplification over a wide range of concentrations (60 ng/µl down to 0.05859 ng/µl with 6 different concentrations in total: Appendix Figure S3 and Figure S4) giving a linear dynamic range of 0.05859 to 60 ng/µl. The reaction was considered consistent when the linear correlation coefficient exceeded 0.985. The efficiency of the telomere amplification was 1.17 and the efficiency of the 18S amplification was 0.97, and samples all fell within the same concentration range as our standards. All runs included the same “golden standard” and also a no‐template control to detect contamination. LinRegPCR 2016.0 (Heart Failure Research Centre, Ruijter et al., 2009, Tuomi, Voorbraak, Jones, & Ruijter, 2010) was used to analyse the qPCR data. The starting concentrations of telomere (T) and control gene (S; 18S) as determined with LinRegPCR were used to determine the relative telomere length with the calculation T/S. Telomeres and the control gene were assessed in separate runs. The mean interassay coefficient of variation for qPCR runs for telomere (*n* = 4) and 18S (*n* = 4) amplification were 2.98% and 0.59%, respectively, calculated using the golden standard. The intra‐assay coefficient of variation for telomere and 18S runs were 1.09% and 0.90%, respectively. A general linear model found no significant difference in the distribution of the sexes among the plates (*F*_1,23_ = 1.211, *p* = 0.283). To investigate possible run effects, we took the mean value for each triplicate as produced by LinRegPCR for the blood data to simplify the analysis. An ANOVA of the ln‐transformed (for normality) 18S data showed no significant difference among the runs (*F*_3,18_ = 0.795, *p* = 0.513) and neither did the runs containing telb (*F*_3,18_ = 0.888, *p* < 0.466).

### 2.4 Statistical analyses

Analyses were conducted with SAS 9.4 (SAS Institute, Cary) and SPSS 25.0 (IBM, Armonk). RTL was ln‐transformed in order to conform to normality, as verified with a Shapiro–Wilk test. Potential effects of body condition (BCI) were assessed by generating the residuals of a regression analysis of mass versus SVL at death. First, to test whether incubation at different temperatures had affected the results, a mixed model analysis of the relationship between RTL and incubation temperature, with individual ID and maternal ID as random factors, was conducted and found not significant (*F*_1,15.1_ = 0.17, *p* = 0.6818, *n* = 78). To further investigate potential effects of incubation temperature, Pearson's correlation coefficients of temperature, incubation time, and hatching mass were calculated. Pearson's correlations were conducted between RTL and SVL at hatching and death, mass at hatching and death, residual BCI at death, age at death, and growth rate (calculated as the difference between hatching and death SVL, divided by age). Pearson's correlation coefficients were also calculated for the telomeres of all combinations of the tissue types to test for similarity in telomere dynamics across the tissues. We chose not to apply a correction (e.g., Bonferroni) to this analysis despite the number of correlations tested for as the low sample sizes available limit our statistical power and increase the probability of type II errors. Application of a correction would only further increase the chance of type II errors and reduce our probability of detecting real effects (Nakagawa, 2004). To test for sex‐ and organ‐specific telomere effects a mixed model analysis of the relationship between RTL and sex, organ type and a sex×organ type interaction was tested with ID included to control for multiple measures from the same individual. The sex×organ type interaction was not significant (*F*_4,73_ = 1.206, *p* = 0.316) but was retained in the final model as it resulted in a smaller −2 Res log likelihood. As organ type effects were detected, pairwise contrasts between each organ type were conducted. Sequential Bonferroni adjustments were performed for all pairwise contrasts. As sex‐based differences in RTL were found, GLMs testing sex‐based differences in SVL at hatching and death, mass at hatching and death, residual BCI at death, age, and growth rate were performed to determine whether size differences might account for the variation in RTL.

## 3 RESULTS

### 3.1 Effects of incubation temperature

Incubation temperature negatively correlated with incubation time (*r* = −0.8039, *n* = 24, *p* < 0.001) and hatchling mass (*r* = −0.5519, *n* = 24, *p* = 0.0052) but not hatchling SVL (*r *= −0.3587, *n* = 24, *p* = 0.0852).

### 3.2 Comparisons among organ types.

**Figure 2:**
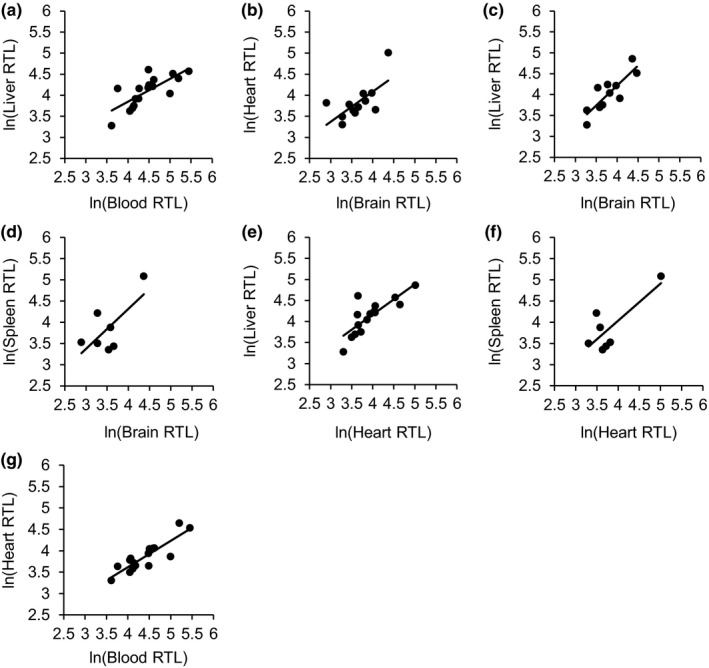
Significant Pearson's correlations between the ln‐transformed relative telomere lengths (RTL) of the various tissue types collected from each individual. a) Correlation between liver and blood RTL (*r* = 0.74517, *p* = 0.0006, *n* = 17). b) Correlation between heart and brain RTL (*r* = 0.66747, *p* = 0.0177, *n* = 12). c) Correlation between liver and brain RTL (*r* = 0.84518, *p* = 0.0011, *n* = 11). d) Correlation between spleen and brain RTL (*r* = 0.68669, *p* = 0.0410, *n* = 9). e) Correlation between liver and heart RTL (*r* = 0.80095, *p* = 0.0003, *n* = 15). f) Correlation between spleen and heart RTL (*r* = 0.73080, *p* = 0.0253, *n* = 9). g) Correlation between heart and blood RTL (*r* = 0.87707, *p* < 0.0001, *n* = 16)

The mixed model analysis (*F*_9,73_ = 4.632, *p* < 0.001) revealed significant differences in RTL among organ types (*F*_4,73_ = 6.964, *p* = 0.003). The dragons had a mean (±*SEM*.) blood RTL of 95.41 (±10.89), a mean heart RTL of 50.65 (±7.641), a mean liver RTL of 63.77 (±6.079), a mean brain RTL of 47.90 (±7.857), and a mean spleen RTL of 43.69 (±12.80). Blood RTL was significantly greater than heart RTL (*t*_73_ = 3.406, *p* = 0.01), brain (*t*_73_ = 4.772, *p* < 0.001), and spleen RTL (*t*_73_ = 3.399, *p* = 0.01). Liver RTL was significantly greater than brain RTL (*t*_73_ = 2.950, *p* = 0.03). Several significant correlations were found between the organ types (see Table 1 and Figure 2).

**Table 1:** Pearson's correlation coefficients for the relative telomere lengths of the different cell types

		Blood	Brain	Heart	Liver	Spleen
Blood	*r*	1.00000	0.38539	0.87707	0.74517	0.59559
	*p*		0.1736	**<0.0001**	**0.0006**	0.0906
	*n*	22	14	16	17	9
Brain	*r*		1.00000	0.66747	0.84518	0.68669
	*p*			**0.0177**	**0.0011**	**0.0410**
	*n*		16	12	11	9
Heart	*r*			1.00000	0.80095	0.73080
	*p*				**0.0003**	**0.0253**
	*n*			17	15	9
Liver	*r*				1.00000	0.68344
	*p*					0.0617
	*n*				18	8
Spleen	*r*					1.00000
	*p*					
	*n*					10

Note

Calculations were performed on ln‐transformed data. Significant correlations (*p* < 0.05) are bolded.

### 3.3 Sex‐based effects on telomere length

**Figure 3:**
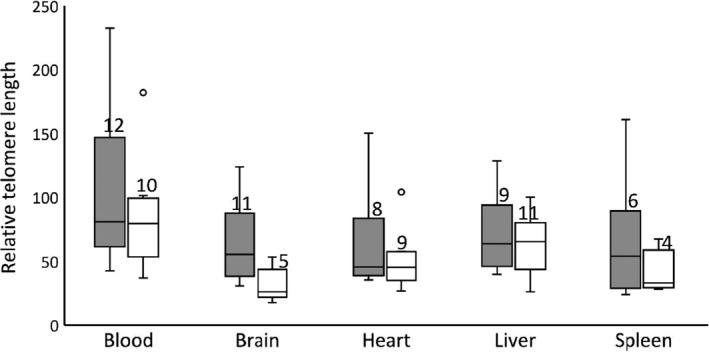
Comparison of the median and interquartile range (IQR, whiskers are minimum and maximum values, outliers are more than 1.5 times the IQR away from the third quartile) of female (gray) and male (white) relative telomere lengths (RTL). Ultimately, 22 blood, 20 liver, 17 heart, 16 brain, and 10 spleen samples from 24 dragons were used in the study. Mixed model analyses showed females had longer telomeres overall (*F*_1,73_ = 9.268, *p* = 0.003), and *t* tests showed female brain telomeres were longer than male brain telomeres (*t*_73_ = 3.227, *p* = 0.002, with Bonferroni adjustment applied). Statistical calculations were performed on ln‐transformed data

Mixed model analyses revealed a significant difference in RTL between the sexes (*F*_1,73_ = 9.268, *p* = 0.003, Figure 3), with females having longer telomeres. Female brain telomeres were approximately twice that of males (*t*_73_ = 3.227, *p* = 0.002) with a RTL approximately twice that of males. No significant body‐size differences at either hatching or death were detected between males and females.

## 4 DISCUSSION

We observed an overall consistency in telomere length (TL) among the organs of juvenile dragons, with females having slightly longer telomeres than males. However, our most striking result is that male lizards had very short telomeres in their brain cells. As these measurements represent a single time point, it is unclear whether males experience rapid telomere attrition after, or even prior to, hatching, or whether females maintain telomere length through telomerase activity. We did not measure the mass of the brain, but doubt that sex‐specific differences in growth rate of the brain would be high enough to explain this discrepancy in TLs (given the low size dimorphism of the species, and the lack of difference in mass or SVL between the sexes in the present study). A more likely explanation, given that males must learn quickly in order to effectively patrol their territories, is that rapid brain development increases metabolic rate and thus ROS production. Investigations of tissue‐ and organ‐specific ROS and antioxidant production could test this hypothesis. Regardless, this result indicates that telomere dynamics can vary greatly between tissues depending upon life history strategy.

We unfortunately do not know the morphs of the male dragons as they had not begun to produce their head colors. This may mean that our dataset was biased if males of a particular morph were overrepresented. Given the differences in behavior and reproductive tactics between red and yellow male dragons, we might predict differences in their brain telomeres also. Red dragons are aggressive, have high testosterone, and outcompete yellow dragons in combat bouts (Healey et al., 2007; Olsson, Healey, & Astheimer, 2007), whereas yellow dragons outcompete red dragons in sperm competition (Olsson et al., 2009). The aggression in red males may be facilitated by rapid MPON development, suggesting that red males may have shorter brain telomeres than yellow males. We have also previously found that red males have shorter blood telomeres than yellow males (Rollings, Friesen, et al., 2017). While in the present study we have only found an indirect correlation of blood and brain telomere length (i.e., blood and brain TL both correlate with heart and liver TL), it is possible that red males may have shorter brain telomeres than yellow males. Given the different life history strategies and reproductive tactics contained within a single species, further research into the males of this species may reveal trends in morph‐specific life history strategies and organ‐specific telomere dynamics.

As females had longer telomeres than males, our results match several previous studies comparing TL between the sexes (Barrett & Richardson, 2011; Rollings, Uhrig, et al., 2017). However, most of these studies were performed on adult organisms, where the trade‐off of resource allocation toward reproduction may have initiated telomeric attrition (although see Schmidt et al., 2016 for tissue comparisons of gull embryos and juveniles). Our results show that the reproductive tactics of the male dragons may present costs prior to maturation. To clarify the generality of this phenomenon, future research needs to focus on a broader range of life stages in organisms to measure the costs associated with particular life history strategies across the entire life span.

Our results suggest that measurements of TL in the blood may serve as a useful proxy for TL in other tissues in dragons. Blood TL positively correlated with heart and liver TL and approached significance with spleen TL, despite the small sample size (*n* = 9). It is unclear why TLs in blood and brain of the same animal were not significantly correlated with each other. The lack of correlation is unlikely to be explained by the unusual telomere dynamics in the brain, as brain TL correlated with heart, liver, and spleen. Unfortunately, low sample size prevents testing for sex‐specific differences in the correlation between blood and brain TL.

Telomeres in the blood were significantly longer than those in the brain, heart, and spleen. This contrasts with other studies which have reported either no difference or shorter blood telomeres compared with other tissues (Benetos et al., 2011; Daniali et al., 2013; Reichert et al., 2013). Little is known about the blood telomeres of reptiles which may simply be longer than the telomeres of other cell types, tissue‐specific telomere lengths in reptiles require further investigation. Alternatively, the blood telomere length may be influenced by the methodology that we used. The much longer incubation used to fully lyse the cells in the organ samples may have caused more damage to the telomeres than to the reference gene due to their location on the chromosome, resulting in shorter telomere measurements. Further investigation is required to determine whether the methodology has influenced the results.

The correlations between tissues must be treated with some caution, as qPCR is unable to exclude interstitial telomeres, which we would predict to be consistent in length across tissues, and may artificially strengthen the relationships (Foote, Vleck, & Vleck, 2013). However, the lack of correlation between blood and brain TL, potentially caused by the difference in brain TL between the sexes, suggests that the observed significant correlations are caused by actual terminal telomeres. As we expect interstitial telomeres to remain consistent between cells, if interstitial telomeres were causing the correlations between tissues we should expect a significant correlation between blood and brain TL, despite potential sex‐based differences. The lack of correlation between blood and brain telomeres gives us confidence that these correlations accurately indicate trends between the tissue types.

Our results provide some support for usage of blood TL as a proxy for the TL of other, harder to gather, and destructively sampled, tissues. However, correlations may not occur under all circumstances. We observed that telomere dynamics may vary between tissues and sexes and may potentially be driven by the life history strategy of the species. While it may not always be possible or practical to compare blood and organ telomeres in a species, care should be taken to consider whether blood telomeres are likely to have the same dynamics as the telomeres of organs of interest. Careful consideration of life history strategies is vital for broadening our understanding of the mechanisms underlying telomere dynamics.

## CONFLICT OF INTEREST

The authors have no conflict of interest to declare.

## AUTHOR CONTRIBUTIONS

Conceptualization: N.R., C.R.F, M.O.; Methodology: N.R., C.R.F, C.M.W, Investigation: N.R., C.R.F, R.J.; Data analysis: N.R., C.R.F., R.S., M.O.; Writing—original draft: N.R.; Writing—review & editing: N.R., C.R.F, C.M.W., R.J., R.S., M.O.; Supervision: C.R.F, C.M.W., R.S., M.O.

## DATA AVAILABILITY

Data is archived in Dryad (https://doi.org/10.5061/dryad.k2t648q).
