# Supplementary material for: Sex‐ And tissue‐specific differences in telomere length in a reptile
Source: Ecol Evol. 2019 May 22;9(11):6211–9. doi: 10.1002/ece3.5164 (PMC6580261; doi:10.1002/ece3.5164)
Supplement: Supplementary file 1 [file ECE3-9-6211-s001.docx]

Supplementary Information


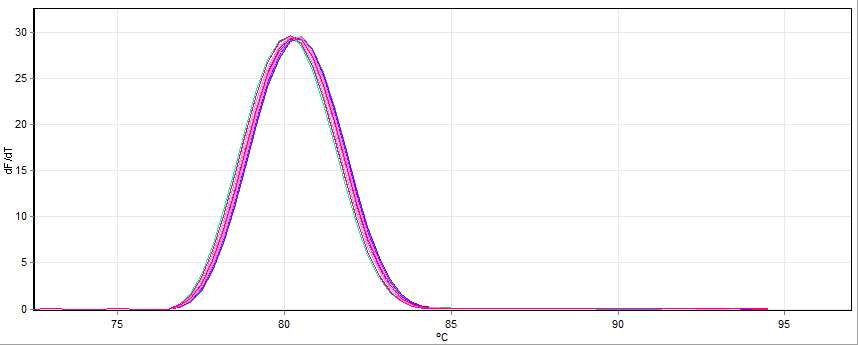


Supplemental figure S1: 18S melt curve, showing the amplification of a single product


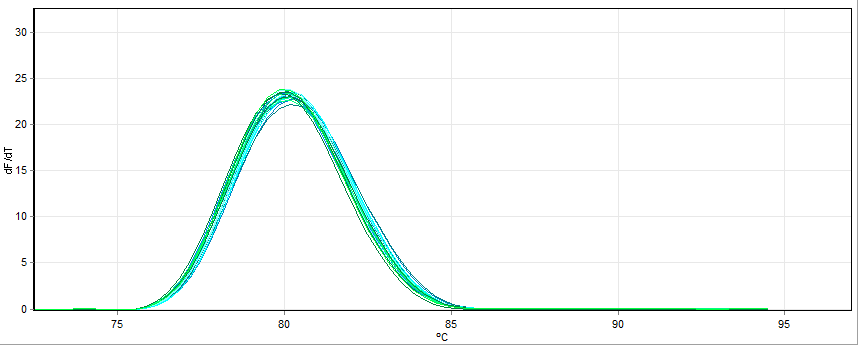


Supplemental figure S2: Telomere melt curve, showing the amplification of a single product
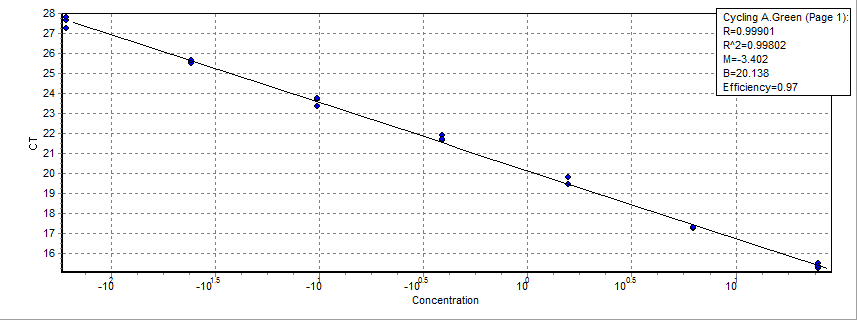


Supplemental figure S3: Standard curve for 18S with R^2^ = 0.99802 and an efficiency of 0.97


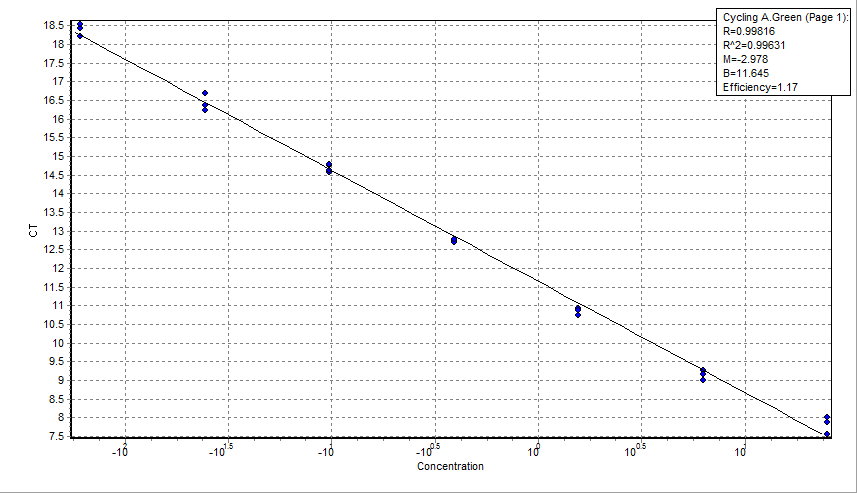


Supplemental figure S4: Standard curve for telomeres with R^2^ = 0.99631 and an efficiency of 1.17
